# Impact of climate events, pollution, and green spaces on mental health: an umbrella review of meta-analyses

**DOI:** 10.1017/S0033291722003890

**Published:** 2023-02

**Authors:** Pim Cuijpers, Clara Miguel, Marketa Ciharova, Manasi Kumar, Luke Brander, Pushpam Kumar, Eirini Karyotaki

**Affiliations:** 1 Department of Clinical, Neuro and Developmental Psychology, Amsterdam Public Health Research Institute, Vrije Universiteit Amsterdam, Amsterdam, the Netherlands; 2 International Institute for Psychotherapy, Babeș-Bolyai University, Cluj-Napoca, Romania; 3 Brain and Mind Institute, Aga Khan University, Nairobi, Kenya; 4 Institute for Environmental Studies, Vrije Universiteit Amsterdam, Amsterdam, the Netherlands; 5 United Nations Environment Programme, Washington, DC, USA

**Keywords:** Anxiety, climate change, depression, green spaces, mental health, pollution, suicide

## Abstract

Climate change may affect mental health. We conducted an umbrella review of meta-analyses examining the association between mental health and climate events related to climate change, pollution and green spaces. We searched major bibliographic databases and included meta-analyses with at least five primary studies. Results were summarized narratively. We included 24 meta-analyses on mental health and climate events (*n* = 13), pollution (*n* = 11), and green spaces (*n* = 2) (two meta-analyses provided data on two categories). The quality was suboptimal. According to AMSTAR-2, the overall confidence in the results was high for none of the studies, for three it was moderate, and for the other studies the confidence was low to critically low. The meta-analyses on climate events suggested an increased prevalence of symptoms of post-traumatic stress, depression, and anxiety associated with the exposure to various types of climate events, although the effect sizes differed considerably across study and not all were significant. The meta-analyses on pollution suggested that there may be a small but significant association between PM_2.5_, PM_10_, NO_2_, SO_2_, CO and mental health, especially depression and suicide, as well as autism spectrum disorders after exposure during pregnancy, but the resulting effect sizes varied considerably. Serious methodological flaws make it difficult to draw credible conclusions. We found reasonable evidence for an association between climate events and mental health and some evidence for an association between pollution and mental disorders. More high-quality research is needed to verify these associations.

## Introduction

It is unequivocal that increasing human activities have led to warming the atmosphere, ocean, and land. Widespread and rapid changes in the atmosphere, ocean, cryosphere, and biosphere have occurred (Legg, 2021). The scale of the recent changes across the climate system is unprecedented over many centuries to many thousands of years (Legg, 2021). These changes are suggested to have serious consequences for human health, including renal function loss, dermatological malignancies, tropical infections, pregnancy complications, allergies, and cardiovascular and pulmonary morbidity and mortality (Atwoli et al., 2021; Haines & Ebi, 2019; Rocque et al., 2021).

Over the last few years it has been suggested that mental health may also be affected by these rapidly occurring climate changes. A recent seminal review describes how mental health may be affected by climate change through four different pathways (Clayton, 2021). First, discrete events, such as natural disasters can have a direct impact on mental health. There is suggestive evidence that floods, heatwaves, tornados and hurricanes, wildfires, and earthquakes may be associated with increased rates of post-traumatic stress and depression, substance use disorders, suicidal thoughts, and important risk factors, such as domestic abuse (Cénat, McIntee, & Blais-Rochette, 2020; Chan & Rhodes, 2014; Chen & Liu, 2015; Dai et al., 2016). Second, mental health can be affected by gradual changes, such as rising sea levels and higher temperatures. Although the causal mechanisms are not clear, higher temperatures, for example, have been associated with more aggression and higher suicide rates (Clayton, 2021). Pollution and the ‘greenhouse’ effect associated with the burning of fossil fuels may also have consequences for mental health. Third, climate change may affect existing physical and social systems, and these changes may have an indirect effect on mental health. For example, the occupational structure and agricultural conditions may change in communities, resulting in economic uncertainties for some groups. Migration may also be the result of areas becoming less inhabitable or disappearing altogether (Clayton, 2021). The fourth pathway refers to the perception of climate change, with anecdotal reports of ‘climate anxiety’, for example in parents who are worried about their children's future and young adults who are reluctant to procreate because of fear about the future (Clayton, 2021). Solastalgia, the distress caused by the transformation and degradation of one's home environment, is a comparable phenomenon that is somewhat better examined, although it is still mostly unclear what it exactly is and how it affects mental health (Galway, Beery, Jones-Casey, & Tasala, 2019). If climate change does indeed have impact on mental health, it is important to include this in the future projections of global mental health and, if possible, measures should be taken to prevent or treat these increasing numbers of patients with mental disorders.

Over the past decade, the number of meta-analyses of studies examining the impact of climate change on mental health has increased exponentially. That is a positive development, as replication increases knowledge about the impact of climate change. However, the published meta-analyses all have a specific focus, for example on one mental health problem, such as posttraumatic stress disorder (PTSD) (e.g. Chan & Rhodes, 2014; Dai et al., 2016; Rezayat et al., 2020), suicide (e.g. Jahangiri, Yousefi, Mozafari, & Sahebi, 2020), or autism spectrum disorders (ASDs) in children (Chun, Leung, Wen, McDonald, & Shin, 2020; Dutheil et al., 2021). Other meta-analyses focus on specific events (e.g. Chen & Liu, 2015; Dai et al., 2016), on one specific country (e.g. Hosseinnejad et al., 2022; Sepahvand, Hashtjini, Salesi, Sahraei, & Jahromi, 2019), or one polluting substance (Forns et al., 2020). Because of the focus on specific subjects, an overview of the whole field is lacking. Furthermore, an increasing number of meta-analyses is not always positive, because it is not uncommon for meta-analyses on the same research question to reach different conclusions, even when published within the same year (Solmi, Correll, Carvalho, & Ioannidis, 2018). Such discrepancies unavoidably lead to confusion and debate among policy makers and researchers (Solmi et al., 2018). Umbrella reviews – a systematic review of all systematic reviews and/or meta-analyses on a given topic – allow a higher-level synthesis of the evidence and a better recognition of the uncertainties, biases, and knowledge gaps, compared to single meta-analyses (Ioannidis, 2009, 2017). An umbrella review on environmental and climate-related determinants of mental health is important, because when it is found that mental health is indeed affected, this would be an additional reason to stop the warming of the atmosphere, ocean, and land as soon as possible.

We conducted an umbrella review of meta-analyses examining environmental and climate-related determinants of mental health. In this review, we focused on three major environmental and climate-related determinants that (1) potentially have a major impact on mental health; (2) represent different, non-overlapping domains, and (3) cover as much as possible of the possible environmental and climate-related determinants.

We defined climate events as ‘discrete episodes of extreme weather or unusual climate conditions, often associated with deleterious impacts on society or natural systems, defined using some metric to characterize either the meteorological characteristics of the event or the consequent impacts’ (Stott et al., 2016). We also used the taxonomy of climate events developed by Stephenson, Diaz, and Murnane (2008), including tropical cyclones, hurricanes, extratropical cyclones, convective phenomena (including tornadoes and severe thunderstorms), mesoscale phenomena (such as polar lows, resulting in e.g. extreme wind speeds and precipitation), floods, drought, heat waves, cold waves, and fog. In this study we also included natural events that may not be directly influenced by the climate such as landslides. Pollution was defined as the addition of any substance (solid, liquid, or gas) or any form of energy (such as heat, sound, or radioactivity) to the environment at a rate faster than it can be dispersed, diluted, decomposed, recycled, or stored in some harmless form (Nathanson, 2022). The major kinds of pollution, usually classified by environment, are air pollution, water pollution, and land pollution. The impact of green spaces was operationalized as the impact of exposure to the natural environment on mental health problems (Roberts, van Lissa, Hagedoorn, Kellar, & Helbich, 2019).

The domains we choose were also relevant for a separate report by the United Nations Environment Programme (UNEP) (in preparation), that included a modeling study examining the impact of these domains on the costs of global mental health. The three specific subjects that are examined in this umbrella review are (1) the impact of climate events on mental health; (2) the impact of pollution on mental health; and (3) the association between green spaces and mental health, as an indicator of the impact of urbanization on mental health.

## Methods

### Search strategy and selection criteria

We used an umbrella review methodology to systematically collect and review all available meta-analyses examining the potential association between climate change, pollution, green spaces, and mental health. We followed general guidelines for conducting and reporting umbrella reviews (Ioannidis, 2009, 2017; Papatheodorou, 2019; Solmi et al., 2018). The protocol for this meta-analysis was registered at the Open Science Framework (Cuijpers, 2021).

We conducted systematic searches on 17 June 2021 in three bibliographic databases: PubMed, PsycINFO, and Embase. We first developed a general search string for climate change, pollution, and green spaces, in which we combined text and key words for these subjects with text and key words for mental health and mental disorders. We limited the results to systematic reviews and meta-analyses. Because the impact of climate events (such as tornados, landslides, heatwaves, etc.) may not be captured by key words related to climate change, we conducted separate searches for these events. In these searches we combined text and key words for climate events with text and key words for mental health and mental disorders and again limited the results to meta-analyses and systematic reviews. The full search strings are available in online Supplement S1. All records were read by two independent researchers and we retrieved the full-text of all studies that were selected for retrieval by one or both researchers.

In this umbrella review we included (a) meta-analyses that reported (b) the association between climate events, green spaces, or pollution and mental health or mental disorders, and (c) in which at least one analysis included more than five comparisons (in order to have a reasonable impression of the association). We included any kind of climate event and pollution as defined in the Introduction, including specific substances, and pollution of air, soil, and water. Any mental health problem or mental disorder was included. Because of limited resources, we excluded studies on the association between climate change and intelligence, as well as on dementia and cognitive decline. Only studies in English were included. No other exclusion criteria were applied.

All full text papers were read by two independent researchers and the decision to include or exclude was based on consensus. Disagreements were solved through discussion. Because no disagreements remained after the discussions, it was not needed to consult a third, senior author.

### Quality assessment

The quality of the included meta-analyses was assessed with the AMSTAR 2, a critical appraisal tool for systematic reviews (Shea et al., 2017). AMSTAR-2 critically assesses 16 core characteristics of systematic reviews (online Supplement S2). All assessments of these criteria were conducted by two independent researchers and disagreements were solved by discussion or, when needed, discussed with a third reviewer.

### Data extraction

We extracted the following data from the included meta-analyses: the examined climate-related factor (climate event, pollution, specific substance, green spaces, etc.), the design of the included studies in the meta-analysis, the number of included studies, the aggregated number of participants in the primary studies (when reported), the population, and the instrument used to measure the quality of primary studies. We also extracted the mental health outcome, a summary of the pooled outcomes, the significance of the outcomes, the level of heterogeneity [*I*^2^ and its 95% confidence interval (CI)], and (when reported) the outcomes of the analyses examining publication bias. When *I*^2^ or its 95% CI were not reported, we calculated them with the value of χ^2^ and degrees of freedom (if available), using the Heterogi module in STATA SE (version 16.1 for Mac). The general characteristics of the meta-analyses were extracted by one reviewer. The outcomes were extracted by one reviewer whose extraction was validated by a second reviewer, who independently extracted 25% of the data. An agreement index of 96.3% between the two reviewers was reached.

### Integration of findings

We offer a narrative overview of the identified associations between indicators for climate change, pollution, and green spaces and indicators for mental health and mental disorders.

## Results

### Selection and inclusion of studies

**Fig. 1.**
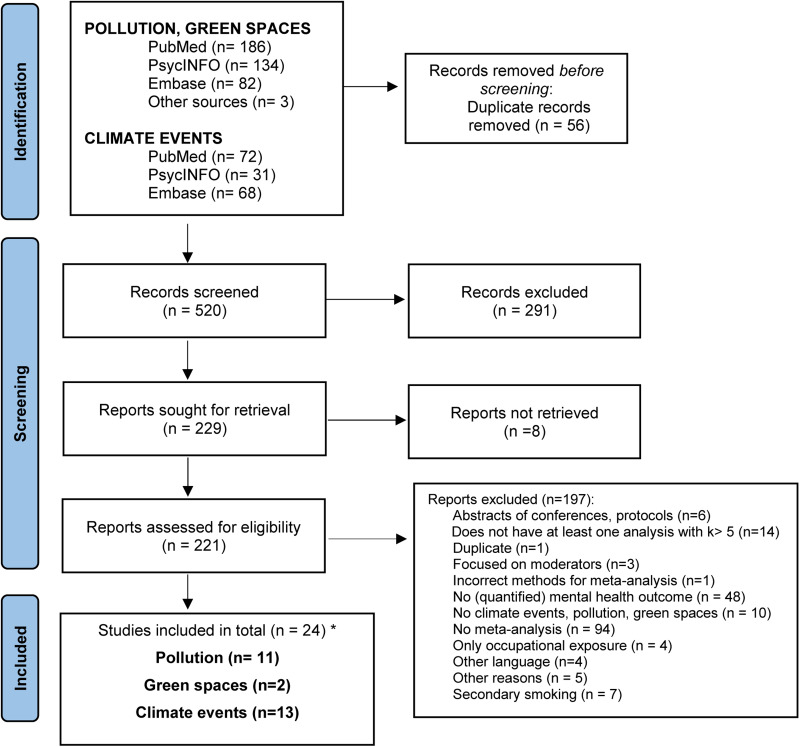
PRISMA flowchart for the inclusion of studies. *One study (Heo et al., 2021) was included in both pollution and climate events, and another study (Generaal et al., 2019) was included in both pollution and green spaces.

We examined the abstracts and titles of 575 records (519 after removal of duplicates). The full-texts of 221 studies were retrieved and assessed for eligibility, from which 197 were excluded. A total of 24 meta-analyses met the inclusion criteria, two for green spaces and natural environments, 11 for pollution, and 13 for climate events (with two meta-analyses included in multiple categories). The PRISMA flowchart is presented in Fig. 1.

### Characteristics of included meta-analyses

The main characteristics of the 24 included meta-analyses are summarized in Table 1. All identified reviews were relatively recent, published until 2021 and with searches covering from databases' inception to 2021. The total number of primary studies included in the meta-analyses ranged from 5 to 64. The total sample sizes varied among meta-analyses, with the largest analysis including 758 997 participants. The most frequently used design in the included primary studies was cross-sectional, but other common designs included case-control studies, cohorts, case-crossover designs, and time-series analyses. Two of the included studies were not systematic reviews, but performed meta-analyses on outcome data from large European (Forns et al., 2020) and Dutch cohorts (Generaal et al., 2019). More details of the included meta-analyses are reported in online Supplement S3.

**Table 1.** Selected characteristics of included meta-analyses

Study	Aims	Exposure	Included studies	Design primary studies	Population	MH outcome	Searches
*Climate events*							
Beaglehole et al. (2018)	Effects of natural disasters on mental health.	Earthquakes, floods, hurricanes, tsunamis, wildfires, etc.	29 in SR, 19 in MA	Exposed *v.* non-exposed (*n* = 8), pre-post (*n* = 8)	Adults	Psychiatric disorders and distress	1980–2017
Cénat et al. (2020)	Prevalence of PTSD, depression, anxiety symptoms, plus other mental health problems among survivors.	Earthquake in Haiti	50 in SR, 28 in MA	Cross-sectional (*n* = 27), longitudinal (*n* = 1)	All ages	PTSD, depression, anxiety	2010–2019
Chan and Rhodes (2014)	Association exposure severity and PTSD.	Hurricane Katrina	8 in MA	Observational (not specified)	Adults	PTSD	2005–2011
Chen and Liu (2015)	Incidence of PTSD among flood victims.	Floods	14 in MA	Observational (not specified)	All ages	PTSD	1980–2014
Dai et al. (2016)	Incidence of PTSD among survivors after earthquakes.	Earthquakes	46 in MA	Cross-sectional (*n* = 40), longitudinal (*n* = 6)	All ages	PTSD	Inception to 2015
Heo et al. (2021)^a^	Suicide risks after short-term exposure to ambient temperature and air pollution.	Temperature (°C)	50 in SR, 18 in MA	Time-series analysis and case-crossover designs	All ages	Suicide (attempt, complete, self-harm, ideation)	Inception to 2020
Hosseinnejad et al. (2022)	Prevalence of PTSD after earthquake in Iran and Pakistan.	Earthquake in Iran and Pakistan	16 in SR, 11 in MA	Cross-sectional (*n* = 10), longitudinal (*n* = 1)	All ages	PTSD	Inception to 2019
Jahangiri et al. (2020)	Prevalence of post-earthquake suicidal ideation.	Earthquakes	8 in MA	Not specified	All ages	Suicide risk (ideation)	2014–2019
Liang et al. (2021)	Occurrence of PTSD after earthquakes among the elderly.	Earthquakes in China	10 in MA	Case-control studies	Older adults	PTSD	2000–2018
Liu et al. (2021)	Effects of high ambient temperatures plus heatwaves on mental health-related mortality and morbidity.	High ambient temperatures and heatwaves	53 in SR, 41 in MA	Time-series (*n* = 32) case-crossover (*n* = 8), case-series (*n* = 1)	All ages	Schizophrenia, substance, mood, neurotic, anxiety disorders	1990–2020
Rezayat et al. (2020)	Prevalence of PTSD among children and adolescents, after earthquakes and floods.	Earthquakes (*n* = 57), floods (*n* = 2)	59 in SR, 39 in MA	Observational (not specified)	Children, adolescents	PTSD	1981–2019
Rubens et al. (2018)	Impact of natural disaster exposure on internalizing problems (other than PTSD) and externalizing problems in youth.	Various natural disasters	64 in MA	Observational (not specified)	Children, adolescents	Internalizing (not PTSD) + externalizing problems	Inception to 2017
Sepahvand et al. (2019)	Prevalence of PTSD after disasters and wars in Iran from 2000 to 2015.	Earthquakes in Iran	10 in MA	Cross-sectional studies	All ages	PTSD	2000–2015
*Pollution*							
Braithwaite. et al. (2019)	Association between PM and adverse mental health outcomes in adults.	Air pollution (PM)	22 in SR, 5 in MA	Cross-sectional (*n* = 5)	Adults	Depression, suicide	Inception to 2017
Chun et al. (2020)	Association maternal exposure to outdoor air pollution and ASD in children.	Air pollution (PM, O_3_, NO_2_)	25 in SR, 14 in MA	Case-control (*n* = 12), cohort (*n* = 2)	Children	ASD	2007–2019
Dutheil et al. (2021)	Risk of ASD in new-borns following air pollution exposure during the perinatal period.	Air pollution (PM, NO*_x_*, O_3_, metals, solvents, styrene, PAHs, pesticides)	28 in SR and MA	Case-control (*n* = 22), cohort (*n* = 6)	Children	ASD	Inception to 2020
Flores-Pajot, Ofner, Do, Lavigne, and Villeneuve (2016)	Association between ambient air pollution and ASD.	Air pollution (PM, NO_2_, and O_3_)	13 in SR, 12 in MA	Cohort (*n* = 7), case-control (*n* = 5)	Children	ASD	Inception to 2016
Forns et al. (2020)^b^	Association between early life exposure to PFOS or PFOA, and ADHD in 9 European population-based studies.	PFAS (PFOS or PFOA)	9 in MA	Cohort (*n* = 9)	Children	ADHD	N/A
Generaal et al. (2019)^b,c^	Association between urbanization, socioeconomic, physical, and social neighborhood characteristics are associated with the prevalence and severity of depression.	Air pollution (PM), and noise pollution	8 in MA	Cohort (*n* = 8)	All ages	Depression	N/A
Hegewald et al. (2020)	Risks of road, railway, or aircraft noise-related for depression, and anxiety among adults.	Traffic noise	28 in SR, 23 in MA	Cross-sectional (*n* = 15), case-control (*n* = 7), cohort (*n* = 6)	Adults	Depression, anxiety	Inception to 2019
Heo et al. (2021)^a^	Suicide risks associated with short-term exposure to ambient temperature and air pollution.	Air pollution (PM, O_3_, SO_2_, NO_2_, CO)	50 in SR, 13 in MA	Time-series, case-crossover designs	All ages	Suicide risk (attempt, complete, self-harm, ideation)	Inception to 2020
Lam et al. (2016)	Does developmental exposure to air pollution affect diagnosis of ASD?	Air pollution (PM)	23 in SR, 6 in MA	Case-control (*n* = 5), cohort (*n* = 1)	Children	ASD	Inception to 2014
Wang, Hossain, Sulaiman, and Ren (2019)	The relationship of ASD with inorganic arsenic (iAs) and lead (Pb) exposure	Pollution (inorganic arsenic and lead)	51 in SR, 25 in MA	Case-control studies (*n* = 25)	Children, adolescents	ASD	Inception to 2018
Zeng et al. (2019)	Observational studies on the association between outdoor air pollution and depression	Air pollution (PM, NO_2_, SO_2_, CO, O_3_)	15 in SR, 14 in MA	Cohort (*n* = 9), cross-sectional (*n* = 3), case-crossover (*n* = 3), time-series (*n* = 1)	All ages	Depression	Inception to 2018
*Green spaces*							
Roberts et al. (2019)	The effect of short-term exposure to the natural environment on depressive mood.	Natural environments	33 in SR and MA	Randomized (*n* = 16), non-randomized (*n* = 5) cross-over, parallel groups (*n* = 7), factorial design (*n* = 3), single-group crossover (*n* = 2)	Adults	Depression	Inception to 2018
Generaal et al. (2019)^b^^,^^c^	Association between urbanization, socioeconomic, physical, and social neighborhood characteristics are associated with the prevalence and severity of depression.	Green spaces and urbanization	8 in MA	Cohort (*n* = 8)	All ages	Depression	N/A

ADHD, attention deficit/hyperactivity disorder; ASD, autism spectrum disorder; CO, carbon monoxide; MA, meta-analysis; NO_2_, nitrogen dioxide; NO*_x_*, nitrogen oxide; O_3_, ozone; PAHs, polycyclic aromatic hydrocarbons; PFAS, perfluoroalkyl substances; PFOA, perfluorooctanoic acid; PFOS, perfluorooctane sulfonate; PM, particulate matter; PTSD, posttraumatic stress disorder; SO_2_, sulfur dioxide; SR, systematic review; MH, Mental health.

a This study is included in the categories of Climate events and Pollution.

b This study is not a systematic review, but because they did combine different datasets we decided to include it.

c This study is included in the categories of Pollution and Green spaces.

### Quality of included meta-analyses

**Fig. 2.**
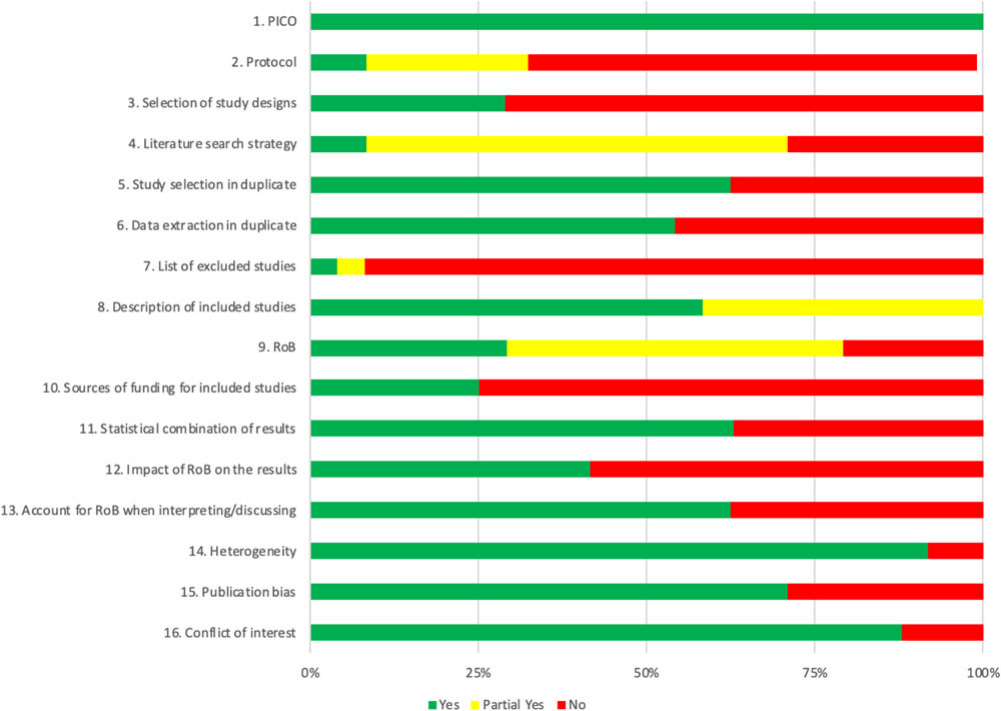
Aggregated AMSTAR-2 ratings for included meta-analyses.

The AMSTAR-2 ratings for each of the 24 included meta-analyses are presented in Table 2, and the aggregated ratings across all reviews are reported in Fig. 2. The specification of PICO was judged as adequate for all studies. The majority of the meta-analyses did not register a protocol (*n* = 16; 67%), did not justify the selection of study designs (*n* = 17; 71%), did not investigate sources of funding (*n* = 18; 75%), and did not provide a list of excluded studies with reasons (*n* = 22; 92%). Comprehensiveness of the literature search was rated as ‘partial yes’ in the majority of reviews (*n* = 15; 63%), while only two reviews obtained a complete positive rating. Two reviewers independently selected studies and extracted data in 15 (63%) and 13 (54%) meta-analyses, respectively. Most of the reviews described in great (*n* = 14; 58%) or sufficient (*n* = 10; 42%) detail the included studies, and investigated sources for heterogeneity (*n* = 22; 92%) and publication bias (*n* = 17; 71%). Above half of them utilized appropriate statistical methods for analyses (*n* = 15; 63%), particularly those reporting on pollution. Risk of bias was adequately assessed in 19 meta-analyses (79%), but only 7 (29%) of these assessed all relevant criteria. The impact of risk of bias on the effect estimates (or inclusion of only studies at low risk) was examined in 10 (42%) meta-analyses, and 15 (62%) accounted for the risk of bias when interpreting or discussing the results. The vast majority of meta-analysts declared their conflicts of interest (*n* = 21; 88%).

**Table 2.** AMSTAR-2 ratings and quality assessment instruments used in the meta-analyses

		AMSTAR-2																
Study	Quality assessment	1	2	3	4	5	6	7	8	9	10	11	12	13	14	15	16	Total^a^
*Climate events*																		
Beaglehole et al. (2018)	NOS (modified)	Y	PY	Y	PY	Y	N	N	Y	Y	N	Y	N	N	Y	N	Y	9
Cénat et al. (2020)	Loney et al.'s (1998) guidelines	Y	PY	N	PY	N	N	N	Y	PY	N	N	Y	Y	Y	N	Y	7.5
Chan and Rhodes (2014)	N/A	Y	N	N	PY	N	N	N	PY	N	N	Y	N	N	Y	Y	Y	6
Chen and Liu (2015)	Loney et al.'s (1998) guidelines	Y	N	N	N	Y	Y	N	PY	PY	N	Y	Y	Y	Y	Y	N	9
Dai et al. (2016)	Loney et al.'s (1998) guidelines	Y	N	N	PY	N	Y	N	PY	PY	N	N	Y	Y	Y	Y	Y	8.5
Heo et al. (2021)	OHAT	Y	N	Y	PY	Y	Y	N	Y	Y	N	Y	N	Y	Y	Y	Y	11.5
Hosseinnejad et al. (2022)	JBI checklist, Hoy et al.'s RoB tool (2012)	Y	N	N	Y	N	N	N	PY	PY	N	N	Y	Y	Y	N	Y	7
Jahangiri et al. (2020)	STROBE	Y	N	N	N	Y	Y	N	PY	PY	Y	N	N	N	Y	Y	Y	8
Liang et al. (2021)	QUADAS-2	Y	N	N	N	N	Y	N	PY	PY	N	N	N	N	Y	Y	Y	6
Liu et al. (2021)	OHAT	Y	Y	Y	N	Y	Y	N	PY	Y	Y	Y	Y	Y	Y	Y	Y	13.5
Rezayat et al. (2020)	NOS	Y	N	N	N	Y	Y	N	PY	PY	N	N	N	N	N	N	Y	5
Rubens et al. (2018)	N/A	Y	N	N	PY	N	Y	N	PY	N	N	Y	N	N	Y	Y	N	6
Sepahvand et al. (2019)	N/A	Y	N	N	N	Y	Y	N	Y	N	N	N	N	N	N	Y	Y	6
*Pollution*																		
Braithwaite et al. (2019)	EPHPP	Y	PY	Y	PY	Y	N	N	Y	PY	N	Y	N	Y	Y	Y	Y	10.5
Chun et al. (2020)	NOS, Lam et al.'s Navig. Guide (2016)	Y	Y	N	PY	N	N	N	Y	Y	N	Y	Y	Y	Y	Y	Y	10.5
Dutheil et al. (2021)	SIGN; NOS	Y	N	N	PY	Y	N	N	Y	PY	N	Y	Y	Y	Y	N	Y	9
Flores-Pajot et al. (2016)	NOS	Y	N	Y	PY	N	N	N	Y	PY	N	Y	Y	Y	Y	Y	N	9
Forns et al. (2020)	N/A	Y	N	N	N	N	N	N	Y	N	Y	Y	N	Y	Y	N	Y	7
Generaal et al. (2019)	N/A	Y	N	N	N	N	N	N	Y	N	Y	Y	N	Y	Y	N	Y	7
Hegewald et al. (2020)	SIGN, CASP	Y	PY	Y	PY	Y	N	Y	Y	PY	Y	Y	Y	Y	Y	Y	Y	13.5
Heo et al. (2021)	OHAT	Y	N	Y	PY	Y	Y	N	Y	Y	N	Y	N	Y	Y	Y	Y	11.5
Lam et al. (2016)	Adapt. Cochrane RoB tool, AHRQ	Y	PY	N	Y	Y	Y	PY	Y	Y	Y	Y	Y	Y	Y	Y	Y	14
Wang et al. (2019)	N/A	Y	N	Y	PY	Y	Y	N	PY	N	N	N	N	N	Y	N	Y	7
Zeng et al. (2019)	NOS	Y	N	N	PY	Y	Y	N	Y	PY	N	Y	N	N	Y	Y	Y	9
Green spaces																		0
Roberts et al. (2019)	Cochrane RoB Tool, ROBINS-I, GRADE	Y	PY	N	PY	Y	N	N	Y	Y	N	N	N	Y	Y	Y	Y	9
Generaal et al. (2019)	N/A	Y	N	N	N	N	N	N	Y	N	Y	Y	N	Y	Y	N	Y	7

a The total is calculated with ‘Y’ counting as 1 point, and ‘PY’ counting as 0.5 points.

Quality assessment instruments: AHRQ, Agency for Healthcare Research and Quality; CASP, Critical Appraisal Skills Programme 2004/2006 assessment tool; EPHPP, Effective Public Health Practice Project; GRADE, Grading of Recommendations Assessment; Development and Evaluation; JBI, Joanna Briggs Institute checklist; NOS, Newcastle–Ottawa Quality Scale; OHAT, OHAT Risk of Bias Rating Tool for Human and Animal Studies; QUADAS-2, Quality Assessment of Diagnostic Accuracy Studies Tool (revised); SIGN, Scottish International Guideline Network checklist; STROBE, Strengthening the Reporting of Observational Studies in Epidemiology Checklist.

AMSTAR-2 items: (1) adequate definition of PICO, (2) methods established before the review, (3) an explanation for the selection of study design to be included, (4) comprehensiveness of the search strategy, (5) study selection by at least two reviewers, (6) data extraction by at least two reviewers, (7) list of excluded studies with reasons, (8) detailed description of included studies, (9) assessment of risk of bias in included studies, (10) reported sources of funding for the included studies, (11) appropriate methods for pooling results, (12) assessment of the impact of risk of bias on the outcomes, (13) discussion of the impact of risk of bias on results, (14) sources of heterogeneity are explored, (15) assessment of publication bias, (16) reporting potential conflict of interest for the review.

The overall confidence in the results was high for none of the studies (zero or one non-critical weakness), for three the confidence was moderate (more than one weakness, but no critical flaws), and for the other studies the confidence was low to critically low. The three studies with moderate confidence were on climate events (Liu et al., 2021) and on pollution (Hegewald et al., 2020; Lam et al., 2016).

### Climate events and mental health

The outcomes reported in the 13 included meta-analyses examining the association between climate events and mental health are summarized in Table 3. We will present the results according to the methodology used, with meta-analyses of pre-post designs first, followed by meta-analyses of estimates of point prevalences of mental disorders (separately for cut-off scores on self-report measures and diagnostic interviews), then meta-analyses of studies with time-series and case-cross-over designs, and finally meta-analyses of correlations between the level of exposure to disasters and mental health outcomes.

**Table 3.** Outcomes of meta-analyses: climate events

Study	Factor	Details of MA	Population	Mental health outcome	*N*	*k*	Estimate	95% CI	*p* value	*I*^2^	95% CI	Publication bias^a^
Beaglehole et al. (2018)	Various natural disasters	Psychological distress after natural disaster	Adults	Psychol. distress	7570	10	SMD = 0.63	0.27–0.98	0.005	98	97–99	NA
		Exposed *v.* non-exposed		Psychol. distress	1968	4	SMD = 1.10	0.23–1.96	0.01	99	98–99	NA
		Pre-post		Psychol. distress	5602	6	SMD = 0.32	−0.06 to 0.70	0.10	99	99–99	NA
		Psychiatric disorders (all combined)		Mental disorders	7043	6	OR = 1.84	1.43–2.38	0.001	76	47–89	NA
		Exposed *v.* non-exposed		Mental disorders	4281	4	OR = 2.14	1.58–2.90	<0.00001	62	0–85	NA
		Pre-post		Mental disorders	2762	2	OR = 1.44	0.98–2.11	0.06	79	NA	NA
		PTSD: exp. *v.* non- exp. (cont.)		PTSD	982	2	SMD = 1.38	0.43–2.34	0.004	97	NA	NA
		PTSD: exp. *v.* non- exp. (binary)		PTSD	1296	2	OR = 5.96	0.25–142.54	0.27	88	NA	NA
		Depression: exp. *v.* non-exp. (cont.)		Depression	1280	4	SMD = 0.90	0.19–1.61	0.01	96	93–98	NA
		Depression: exp. *v.* non-exp. (binary)		Depression	1327	2	OR = 1.29	0.88–1.90	0.20	0	NA	NA
		Depression: pre-post (cont.)		Depression	844	3	SMD = 0.10	−0.04 to 0.23	0.17	0	0–90	NA
		Anxiety: exp. *v.* non- exp. (cont.)		Anxiety	1280	4	SMD = 0.91	−0.08 to 1.90	0.07	98	97–99	NA
		Anxiety: exp. *v.* non-exp. (binary)		Anxiety	1327	2	OR = 1.33	0.91–1.93	0.14	0	NA	NA
		Alcohol dis: exp. *v.* non- exp. (binary)		Alcohol	1327	2	OR = 1.17	0.76–1.79	0.47	0	NA	NA
Cénat et al. (2020)	Earthquakes	Pooled prevalence post-earthquake	All ages	PTSD	7997	24	prop = 0.28	0.18–0.42	NA	99	NA	NA
				Depression	5375	14	prop = 0.32	0.24–0.42	NA	100	NA	NA
				Anxiety	929	5	prop = 0.20	0.16–0.26	NA	67	NA	NA
Chan and Rhodes (2014)	Hurricanes	Association between exposure severity and PTSD	Adults	PTSD	2934	8	*r* = 0.266	0.173–0.355	<0.01	84	69–91	NS (E)
Chen and Liu (2015)	Floods	Combined incidence after a flood	All ages	PTSD	40 600	14	prop = 0.16	0.11–0.21	NA	98	98–99	NS (E)
Dai et al. (2016)	Earthquakes	Pooled incidence of PTSD after earthquake	All ages	PTSD	76 101	46	prop = 0.24	0.19–0.28	NA	100	NA	NS (E)
Heo et al. (2021)	Heat exposure	Suicide rates per IQR increase in temperature (7.1°C)	All ages	Suicide (attempts, complete, self-harm, ideation)		25	RR = 1.09	1.06–1.13	<0.001	97	NA	NS (E)
Hosseinnejad et al. (2022)	Earthquakes	Prevalence of PTSD (after Iran and Pakistan earthquakes)	All ages	PTSD		12	prop = 0.56	0.50–0.61	NA	96	NA	NA
Jahangiri et al. (2020)	Earthquakes	Prevalence of suicidal ideation (4–96 months)	All ages	Suicide ideation	14 347	8	prop = 0.20	0.14–0.27	<0.001	99	NA	NS (E)
Liang et al. (2021)	Earthquakes	Prevalence of PTSD after earthquake	Elderly	PTSD	4834	10	Prop = 0.25	0.20–0.29	NA	92	NA	Susp. (F)
Liu et al. (2021)	Heat exposure	Association high temperatures and MH morbidity (morb.) + mortality (mort.)	All ages	Overall MH (morb.)		12	RR = 1.009	1.006–1.012	NA	78	NA	*p* = 0.002 (E)
				Overall MH (mort.)		5	RR = 1.031	1.011–1.052	NA	72	NA	*p* = 0.007 (E)
				Schizophr. (morb.)		7	RR = 1.007	1.002–1.011	NA	80	NA	NA
				Schizophr. (mort.)		2	RR = 1.008	0.968–1.048	NA	0	NA	NA
				Subst. dis (morb.)		3	RR = 1.008	0.996–1.021	NA	70	NA	NA
				Subst. dis (mort.)		4	RR = 1.046	0.991–1.101	NA	88	NA	NA
				Mood dis (morb.)		6	RR = 1.011	1.003–1.018	NA	87	NA	NA
				Neur. + anx. dis (morb.)		6	RR = 1.007	1.001–1.013	NA	80	NA	NA
				Other (morb.)		3	RR = 1.005	1.001–1.009	NA	18	NA	NA
				Suicides (completed)		7	RR = 1.012	1.003–1.021	NA	80	NA	NS (E)
Rezayat et al. (2020)	Earthquakes and floods	Prevalence of PTSD (⩽6 months after disaster)	Youth	PTSD		21	prop = 0.192	0.186–0.197	<0.001	NA	NA	NA
		After 6–12 months				19	prop = 0.30	0.295–0.306	<0.001	NA	NA	NA
		After 12–18 months				12	prop = 0.244	0.234–0.254	<0.001	NA	NA	NA
		After 12–24 months				6	prop = 0.204	0.191–0.217	<0.001	NA	NA	NA
		Prevalence of PTSD based on diagnosis (⩽6 months after disaster)				NA	prop = 0.208	NA	NA	NA	NA	NA
		6–12 months				NA	prop = 0.146	NA	NA	NA	NA	NA
		12–18 months				NA	prop = 0.163	NA	NA	NA	NA	NA
Rubens et al. (2018)	Various natural disasters	Association between disaster exposure and internalizing problems (other than PTSD)	Youth	Internalizing (no PTSD) problems	376 990	70	*r* = 0.18	0.14–0.22	<0.001	95	94–96	Susp. (F)
		Association between disaster exposure and externalizing problems		Externalizing problems	27 496	31	*r* = 0.08	0.03–0.14	0.002	95	93–96	Not Susp. (F)
Sepahvand et al. (2019)	Earthquakes	Prevalence of PTSD following earthquakes	All ages	PTSD		10	prop = 0.58	0.41–0.75	NA	99	99–99	NS (B)

ADHD, attention deficit/hyperactivity disorder; ASD, autism spectrum disorder; *k*, comparisons included in the analysis; *N*, number of participants; OR, odds ratio; prop, proportion; PTSD, posttraumatic stress disorder; *r*, correlation; RR, risk ratio; SMD, standardized mean difference.

a Publication bias: NS indicates not significant; (E) indicates Egger's test; (B) indicates Begg and Mazumbar's test; Susp. (F) indicates suspected publication bias based on funnel plot inspection; Not Susp. (F) indicates that publication bias is not suspected based on funnel plot inspection; NA indicates not available. Underlined values are significant.

One meta-analysis examined the impact of various natural disasters (earthquakes, floods, hurricanes, tsunamis, wildfires, etc.) on psychological distress and mental disorders, and included studies that compared exposed to non-exposed people, as well as studies with measurements before and after the event (Beaglehole et al., 2018). The studies with both pre-test and post-test assessments are especially informative about the actual number of cases triggered by the events. Six included studies that used a pre-post design found a non-significant standardized mean difference (SMD) of 0.32 (95% CI −0.06 to 0.70) on psychological distress. Only two included studies with a pre-post design were available for measuring the impact on mental disorders, which were too few for providing reliable estimates of the impact.

Eight meta-analyses aimed at examining the pooled prevalence of mental disorders after a climate event (Cénat et al., 2020; Chen & Liu, 2015; Dai et al., 2016; Hosseinnejad et al., 2022; Jahangiri et al., 2020; Liang, Zeng, Liu, Xu, & Liu, 2021; Rezayat et al., 2020; Sepahvand et al., 2019). However, they pooled prevalence rates based on different cut-off values from different self-report measures, which is a crucial methodological error, because these estimates indicate different variables and should not be combined in a meta-analysis (Levis et al., 2019).

Two meta-analyses reported the prevalence of PTSD according to a diagnostic interview. One found a prevalence of diagnosed PTSD after floods of 16% (95% CI 0.11–0.21) (Chen & Liu, 2015), the other reported a prevalence of PTSD in children and adolescents after earthquakes and floods of 20.8% between 0 and 6 months after the event, 14.6% at 6–12 months, and 16.3% at 12–18 months (Rezayat et al., 2020).

Two meta-analyses of mostly time-series designs and case-crossover studies examined the association between heat exposure and mental health problems. One found a significant risk ratio (RR) of 1.09 (95% CI 1.06–1.13) for the association between daily suicide rates and an increase in temperature of 7.1°C (Heo, Lee, & Bell, 2021). The other (Liu et al., 2021) found an overall RR of 1.02 (95% CI 1.01–1.03) for mental health-related mortality for each degree Celsius increase in temperature, and 1.01 (95% CI 1.007–1.015) for mental health-related morbidity. It should be noted that this meta-analysis scored relatively high on AMSTAR-2 (moderate confidence).

Two other studies examined the correlation between the level of exposure to disasters and mental health outcomes. One found a significant correlation between the severity of exposure and PTSD after hurricane Katrina (*r* = 0.27; 95% CI 0.17–0.36) (Chan & Rhodes, 2014). The other found significant correlations for internalizing (*r* = 0.18; 95% CI 0.14–0.22), and for externalizing problems (*r* = 0.08; 95% CI 0.03–0.14) (Rubens, Felix, & Hambrick, 2018).

### Pollution and mental health

The outcomes reported in the 11 included meta-analyses examining the association between pollution and mental health are summarized in Table 4. We will present the results according to the different polluting substances, starting with the most examined substance.

**Table 4.** Outcomes of meta-analyses: pollution and green spaces

Study	Factor	Details of MA	Population	Mental health outcome	*N*	*k*	Estimate	95% CI	*p* value	*I*^2^	95% CI	Publication bias ^a^
*Pollution*												
Braithwaite et al. (2019)	Air pollution	Long-term exposure (⩾6 months) PM_2.5_	Adults	Depression	84 619	5	OR = 1.10	1.02–1.19	0.011	0	NA	NS (E)
		Long-term (⩾6 months) PM_10_	Adults	Depression	38 826	3	OR = 0.89	0.50–1.58	0.692	0	NA	NS (E)
		Short-term (<6 months) PM_10_ (lag 0–1 day)	Adults	Suicide (completed)	24 327	3	RR = 1.01	0.99–1.03	NA	45	NA	NS (E)
		Short-term (<6 months) PM_10_ (lag 0–2 days)	Adults	Suicide (completed)	28 668	4	RR = 1.02	1.00–1.03	0.031	44	NA	NS (E)
Chun et al. (2020)	Air pollution	Exposure during pregnancy to PM_2.5_	Children	ASD		9	OR = 1.06	1.01–1.11	NA	91	NA	NS (E)
		PM_10_				9	OR = 1.01	0.99–1.03	ns	75	NA	NS (E)
		NO_2_				7	OR = 1.02	1.01–1.04	NA	58	NA	NS (E)
		O_3_				4	OR = 1.00	1.00–1.01	ns	55	NA	NS (E)
Dutheil et al. (2021)	Air pollution	Exposure during pregnancy to all air pollutants (PM_10_, NO*_x_*, O_3_, metals, etc.)	Children	ASD	758 997	28	OR = 1.039	1.03–1.05	NA	71	NA	NA
		PM_2.5_				13	OR = 1.11	1.08–1.14	NA	76	NA	NA
		PM_10_				12	OR = 1.00	0.98–1.01	NA	54	NA	NA
		NO*_x_*				12	OR = 1.05	1.03–1.07	NA	70	NA	NA
		O_3_				7	OR = 1.03	1.01–1.05	NA	44	NA	NA
		Metals				7	OR = 1.00	0.95–1.05	NA	68	NA	NA
		Solvents				5	OR = 1.03	1.01–1.05	NA	0	NA	NA
		Styrene				3	OR = 1.32	1.09–1.54	NA	0	NA	NA
		PAHs				3	OR = 1.05	0.83–1.27	NA	30	NA	NA
		Pesticides				3	OR = 0.83	0.68–0.99	NA	21	NA	NA
Flores-Pajot et al. (2016)	Air pollution	Exposure during pregnancy to PM_2.5_	Children	ASD		8	RR = 1.34	0.83–2.17	NA	90	NA	Susp. (F)
		PM_10_				6	RR = 1.03	0.77–1.37	NA	72	NA	NA
		NO_2_				8	RR = 1.05	0.99–1.11	NA	43	NA	NA
		O_3_				2	RR = 1.05	1.01–1.10	NA	0	NA	NA
Generaal et al. (2019)	Air pollution	PM_2.5_	All ages	Depression	32 487	8	OR = 1.07	1.01–1.12	0.02	0	0–62	NA
Heo et al. (2021)	Air pollution	PM_2.5_	All ages	Suicide risk (attempt, complete, self-harm, ideation)		7	RR = 1.02	1.00–1.05	0.098	61	NA	NS (E)
		PM_10_				7	RR = 1.01	1.00–1.03	0.054	62.3	NA	*p* = 0.008 (E)
		O_3_				5	RR = 1.02	0.96–1.10	0.491	54.1	NA	NS (E)
		SO_2_				5	RR = 1.02	1.00–1.04	0.222	71.5	NA	NS (E)
		NO_2_				6	RR = 1.03	1.00–1.07	0.041	64.1	NA	NS (E)
		CO				4	RR = 1.02	0.95–1.08	0.631	61.5	NA	NS (E)
Lam et al. (2016)	Air pollution	Exposure to PM_2.5_ during pregnancy or prior to assessment of ASD	Children	ASD	.	3	OR = 2.32	2.15–2.51	NA	0	NA	NA
		PM_10_			153 554	6	OR = 1.07	1.06–1.08	NA	2.0	NA	NA
Zeng et al. (2019)	Air pollution	PM_2.5_ long-term exposure (>12 months)	All ages	Depression		10	OR = 1.06	1.00–1.13	NA	41.4	NA	NA
		PM_2.5_ short-term (<2 weeks)				4	OR = 1.01	0.99–1.03	NA	69.6	NA	NA
		PM_10_ long-term (>12 months)				8	OR = 1.04	0.85–1.26	NA	75.2	NA	NA
		PM_10_ short-term (<2 weeks)				4	OR = 1.03	1.01–1.05	NA	88.5	NA	NA
		NO_2_ long-term (>12 months)				8	OR = 1.02	0.96–1.09	NA	69.9	NA	NA
		NO_2_ short-term (<2 weeks)				4	OR = 1.04	1.01–1.07	NA	52.0	NA	NA
		O_3_ long-term (>12 months)				6	OR = 1.01	0.99–1.03	0.384	81.3	NA	NA
		SO_2_ short-term (<2 weeks)				2	OR = 1.03	1.00–1.06	0.029	76.8	NA	NA
		CO short-term (<2 weeks)				3	OR = 1.01	1.00–1.01	<0.001	70.2	NA	NA
Forns et al. (2020)	Pollution (perfluoroalkyl substances)	PFOS at birth	Children	ADHD		9	OR = 0.99	0.92–1.07	NA	0	NA	NA
		PFOA at birth				9	OR = 1.01	0.93–1.11	NA	0	NA	NA
		PFOS at 3 months				9	OR = 0.99	0.92–1.06	NA	0	NA	NA
		PFOA at 3 months				9	OR = 1.02	0.93–1.11	NA	0	NA	NA
		PFOS at 24 months				9	OR = 0.97	0.88–1.07	NA	0	NA	NA
		PFOA at 24 months				9	OR = 0.99	0.88–1.12	NA	0	NA	NA
Wang et al. (2019)	Pollution (inorganic arsenic and lead)	Inorganic arsenic in cases (hair concentration, μg/g)	Youth	ASD	168	4	*M* = 0.52	−0.32 to 1.36	Sign. higher (<0.001)	49.4	NA	NA
		Inorganic arsenic in controls (hair concentration, μg/g)			183	4	*M* = 0.10	0.01–0.19		18.9	NA	NA
		Inorganic arsenic in cases (blood concentration, μg/dL)			318	4	*M* = 1.95	−1.01 to 4.91	Sign. higher (<0.001)	99.7	NA	NA
		Inorganic arsenic in controls (blood concentration, μg/dL)			304	4	*M* = 0.37	0.28–0.46		0	NA	NA
		Lead in cases (hair concentration, μg/g)			214	7	*M* = 5.95	0.39–11.51	Sign. higher (<0.001)	99.8	NA	NA
		Lead in controls (hair concentration, μg/g)			262	7	*M* = 2.76	−0.30 to 5.82		99.7	NA	NA
		Lead in cases (blood concentration, μg/dL)			337	7	*M* = 0.93	0.08–1.78	Sign. lower (<0.001)	14.8	NA	NA
		Lead in controls (blood concentration μg/dL)			243	7	*M* = 1.28	0.44–2.12		21.4	NA	NA
		Lead in cases (urine concentration, μg/g)			110	3	*M* = 0.76	−0.78 to 2.29	0.309	0	NA	NA
		Lead in controls (urine concentration, μg/g)			89	3	*M* = 0.90	−1.37 to 3.17		14.8	NA	NA
Hegewald et al. (2020)	Noise	Road traffic	Adults	Depression		11	OR = 1.03	0.99–1.06	NA	60	NA	Susp. (F)
		Aircraft				5	OR = 1.14	1.12–1.15	NA	0	NA	Susp. (F)
		Railway traffic				3	OR = 1.02	0.95–1.08	NA	96	NA	NA
		Road traffic		Anxiety		8	OR = 1.02	0.98–1.06	NA	61	NA	Susp. (F)
Generaal et al. (2019)	Noise	Traffic noise (road, rail, and air traffic)	All ages	Depression	32 487	8	OR = 1.05	0.96–1.15	0.24	46	0–76	NA
*Green spaces*												
Roberts et al. (2019)	Green spaces/nature	Exposure to natural environments	Adults	Depression		40	SMD = 0.30	0.10–0.50	<0.01	86	82–89	Inconclusive
Generaal et al. (2019)	Green spaces/urbanization	Increase of urbanization	All ages	Depression	32 487	8	OR = 1.05	1.01–1.10	0.03	0	0–63	NA
		30% increase of green space in the neighborhood			32 487	8	OR = 0.94	0.88–0.99	0.03	0	0–67	NA

ADHD, attention deficit/hyperactivity disorder; ASD, autism spectrum disorder; CO, carbon monoxide; *k*, comparisons included in the analysis; *N*, number of participants; NO_2_, nitrogen dioxide; NO*_x_*, nitrogen oxide; OR, odds ratio; O_3_, ozone; PAHs, polycyclic aromatic hydrocarbons; PFOA, perfluorooctanoic acid; PFOS, perfluorooctane sulfonate; PM, particulate matter; PTSD, posttraumatic stress disorder; *r*, correlation; RR, risk ratio; SMD, standardized mean difference; SO_2_, sulfur dioxide.

a Publication bias: NS indicates not significant; (E) indicates Egger's test; (B) indicates Begg and Mazumbar's test; Susp. (F) indicates suspected publication bias based on funnel plot inspection; Not Susp. (F) indicates that publication bias is not suspected based on funnel plot inspection.

Particulate matter (PM) is a complex mixture that is usually classified into PM_2.5_ (<2.5 μm in diameter) and PM_10_ (<10 μm) (Chun et al., 2020). One analysis of eight Dutch cohorts found a significant pooled prevalence of depression [odds ratio (OR) = 1.07] per 0.2 × 10^−5^ increase in PM_2.5_ absorbance (Generaal et al., 2019), and two other meta-analyses also reported small but significant associations at the shorter (<6 months) and longer terms (>6 months) (ORs = 1.10 and 1.06) (Braithwaite, Zhang, Kirkbride, Osborn, & Hayes, 2019; Zeng, Lin, Liu, Liu, & Li, 2019). One meta-analysis reported a significant association between depression and PM_10_ at the very short term (<2 weeks) (OR = 1.03) (Zeng et al., 2019).

Other pollutants were found to be significantly associated with depression: nitrogen dioxide (NO_2_; OR = 1.04), carbon monoxide (CO; OR = 1.01); and sulfur dioxide (SO_2_; OR = 1.03). Moreover, a significant association was also found between noise from aircrafts and depression (OR = 1.14).

Several meta-analyses found significant associations between pollutants and ASD when the mothers were exposed during pregnancy: PM_2.5_ (ORs ranging from 1.06 to 2.32), PM_10_ (OR = 1.07), ozone (O_3_; OR = 1.03 and RR = 1.05), NO_2_ (OR = 1.02), and solvents (OR = 1.03). One study found a lower risk for ASD and pesticides (OR = 0.83). Other meta-analyses found a significant association between PM_10_ and suicide (RR = 1.02) and between NO_2_ and suicide (RR = 1.03).

No other significant associations between pollution and mental disorders were found in the included meta-analyses.

### Green spaces and mental health

The results of the two meta-analyses examining the association between green spaces and mental health are reported in Table 4. One pooled analysis of eight Dutch cohort studies found a significant negative association between 30% increase of green space in the neighborhood and depression (OR = 0.94), and a significant association between grade of urbanization (mean number of addresses per km^2^ within a radius of 1 km) and depression (OR = 1.05) (Generaal et al., 2019). The other meta-analysis included a wide range of designs (randomized, non-randomized, parallel groups, etc.), and found a significant effect size (SMD = 0.30) for reduction of depressive mood after short-term exposure to natural environments (Roberts et al., 2019).

## Discussion

We conducted an umbrella review of meta-analyses investigating the association between climate events, pollution, and green spaces on the one hand, and any mental health symptoms or disorders on the other. We included 24 meta-analyses, all relatively recent, which analyzed mainly cross-sectional and case-control studies. The 13 meta-analyses examining the impact of climate events on mental health suggested that there may be an association between such events and mental health, although the exact contribution of these events to the prevalence cannot be established based on these studies. Only two meta-analyses used a diagnostic interview to establish the presence of mental disorders, seven pooled different measures for mental health problems incorrectly, and only six primary studies used a pre-post design, which can be used as a good indication for the impact of climate events on mental health problems. Although it seems obvious that traumatic events like climate events result in increased levels of mental health problems, these methodological problems make it impossible to assess the exact size of the impact.

The 10 meta-analyses focusing on the impact of pollution on mental health, found that some substances are associated with mental health problems, especially depression and ASD in children of mothers exposed to substances. However, all associations were very small and in many studies no corrections were applied for other characteristics of the populations, making these associations highly uncertain. PM_2.5_ was examined in most studies, but other substances that were examined included PM_10_, NO_2_, O_3_, SO_2_, CO, solvents, styrene exposure, and noise. All associations were small and because unmeasured confounders may further reduce the strength and level of significance of the association, these findings should be considered with caution.

We only found two meta-analyses examining green spaces and natural environments (Generaal et al., 2019; Roberts et al., 2019). Both meta-analyses found a small but significant association between green spaces and reduction of mental health symptoms, but the observational nature of many of the primary studies as well as the potential for risk of bias limit confidence in these findings.

Research on the impact of climate events has demonstrated considerable methodological limitations. It is difficult to indicate whether a climate event is caused by climate change or that it is a natural fluctuation. It is also difficult to examine the impact of a climate event on mental health, because this requires at least a measurement before the event and one after, and then the causality is still unclear because other changes may have taken place at the same time that have (partly) caused the changes in mental health. Many studies simply examine correlations between for example pollution and a mental health outcome. Although some studies do adjust for confounders, the number of confounders is usually limited and there is no way to exclude the possibility that an association is in fact caused by a third, unknown variable, especially when the effect size of the identified association is small. Most studies also measured mental health with self-report measures, while such scales cannot reliably indicate the presence of a mental disorder. Studies based on mental disorders assessed with the gold standard, diagnostic interviews, are hardly done, also because the costs and logistic challenges of such studies are considerable. Based on our umbrella review it is not possible to indicate how much support is available for the four different pathways of how climate can have an impact on mental health.

The results of this study need to be counterbalanced weighing new insights it offers and limitations that remain challenging for generalizability of the findings. The strengths include a rigorous approach to umbrella reviews and the inclusion of a considerable number of studies. However, this study also has some important limitations that have to be taken into consideration. One important limitation is that we only included meta-analyses with at least five studies. That threshold is arbitrary because the strength of evidence does not only depend on the number of studies, but also on the size of the study, the design, and the quality. However, we had to limit the scope of this umbrella review to make the rapid scoping of the evidence manageable and we believe that a threshold of five studies does give good indications for the state of research in a particular area. A second important limitation is that the quality of the included meta-analyses was not optimal. The overall confidence in the results was high for none of the studies, and moderate for only three of the 24 included meta-analyses. The confidence in the other meta-analyses was low to critically low. This means that the results of all meta-analyses should be considered with caution because of limited quality. Another limitation is that the impact of climate events, pollution, and green spaces change over time, and while most included meta-analyses were conducted over the past few years, it cannot be examined whether the impact of these changes has increased over time.

This umbrella review shows that very little good evidence is available on the association between mental health on the one hand and climate events, pollution, and green spaces on the other hand. Because of the devastating impact climate events can have on human lives, there should be no doubt that they result in increased mental health problems. However, the current evidence gives no clear indication of how large that increase is. More research with better designs and assessments are clearly needed to get a better overview of the implication of these developments for mental health. This information is needed to develop strategies to reduce the impact of such events on mental health.

In conclusion, in this umbrella review we found some evidence for an association between climate events and mental health, especially post-traumatic stress, but also depression and anxiety. We also found some evidence for an association between pollution and aspects of mental health, especially depression, suicide and autism spectrum disorders. Finally, we found some indications for an association between green spaces and depression. However, more high-quality research is needed to verify all these associations. Given how rapidly the natural world is changing around us and the policy impetus to reflect and revisit human footprint on natural environment, improving design, measurement, and carrying out evidence informed environmental mental health studies would be important.

## Supplementary material

For supplementary material accompanying this paper visit https://doi.org/10.1017/S0033291722003890. click here to view supplementary material
