# Supplementary material for: Impact of climate events, pollution, and green spaces on mental health: an umbrella review of meta-analyses
Source: Psychol Med. 2023 Jan 6;53(3):638–53. doi: 10.1017/S0033291722003890 (PMC9975983; doi:10.1017/S0033291722003890)
Supplement: Supplementary file 1 [file S0033291722003890sup001.docx]

Supplemental materials

Supplement 1. Search strings

A. Searches climate events

**PubMed**

(("climate change"[All Fields] OR ("floodings"[All Fields] OR "floods"[MeSH Terms] OR "floods"[All Fields] OR "flood"[All Fields] OR "flooded"[All Fields] OR "implosive therapy"[MeSH Terms] OR ("implosive"[All Fields] AND "therapy"[All Fields]) OR "implosive therapy"[All Fields] OR "flooding"[All Fields]) OR ("landslides"[MeSH Terms] OR "landslides"[All Fields] OR "landslide"[All Fields] OR "landsliding"[All Fields]) OR "heatwave"[All Fields] OR "heat wave"[All Fields] OR ("droughted"[All Fields] OR "droughting"[All Fields] OR "droughts"[MeSH Terms] OR "droughts"[All Fields] OR "drought"[All Fields]) OR ("tornadoes"[MeSH Terms] OR "tornadoes"[All Fields] OR "tornado"[All Fields] OR "tornados"[All Fields]) OR ("cyclonic storms"[MeSH Terms] OR ("cyclonic"[All Fields] AND "storms"[All Fields]) OR "cyclonic storms"[All Fields] OR "hurricane"[All Fields] OR "hurricanes"[All Fields] OR "hurricane s"[All Fields]) OR ("thunderstorm"[All Fields] OR "thunderstorms"[All Fields]) OR ("cyclone s"[All Fields] OR "cyclonic storms"[MeSH Terms] OR ("cyclonic"[All Fields] AND "storms"[All Fields]) OR "cyclonic storms"[All Fields] OR "cyclone"[All Fields] OR "cyclones"[All Fields] OR "cyclonic"[All Fields]) OR ("avalanches"[MeSH Terms] OR "avalanches"[All Fields] OR "avalanche"[All Fields] OR "avalanching"[All Fields]) OR ("snow"[MeSH Terms] OR "snow"[All Fields] OR "blizzard"[All Fields] OR "blizzards"[All Fields]) OR ("earthquake s"[All Fields] OR "earthquakes"[MeSH Terms] OR "earthquakes"[All Fields] OR "earthquake"[All Fields]) OR ("wildfires"[MeSH Terms] OR "wildfires"[All Fields] OR "wildfire"[All Fields]) OR "coldwave"[All Fields] OR "cold wave"[All Fields]) AND ("mental health"[All Fields] OR "mental disorder"[All Fields])) AND (meta-analysis[Filter] OR systematicreview[Filter])

**Psychinfo**

("climate change" or floods or landslides or "heatwave" or "heat wave" or droughts or tornado or hurricane or thunderstorm or cyclone or avalanche or blizzard or earthquake or wildfire or coldwave or "cold wave") and ("mental health" or "mental disorder”)

**Embase**

'climate change'/exp OR 'climate change' OR 'floods'/exp OR floods OR 'landslides'/exp OR landslides OR 'heatwave'/exp OR 'heatwave' OR 'heat wave'/exp OR 'heat wave' OR 'droughts'/exp OR droughts OR 'tornado'/exp OR tornado OR 'hurricane'/exp OR hurricane OR 'thunderstorm'/exp OR thunderstorm OR 'cyclone'/exp OR cyclone OR 'avalanche'/exp OR avalanche OR 'blizzard'/exp OR blizzard OR 'earthquake'/exp OR earthquake OR 'wildfire'/exp OR wildfire OR coldwave OR 'cold wave'

AND

('meta analysis'/de OR 'systematic review'/de) AND ('addiction'/dm OR 'alcoholism'/dm OR 'antenatal depression'/dm OR 'anxiety disorder'/dm OR 'autism'/dm OR 'depression'/dm OR 'drug dependence'/dm OR 'heroin dependence'/dm OR 'major depression'/dm OR 'mental disease'/dm OR 'phobia'/dm OR 'postnatal depression'/dm OR 'posttraumatic stress disorder'/dm OR 'psychosis'/dm OR 'sleep disorder'/dm OR 'suicidal behavior'/dm OR 'suicide attempt'/dm)

B. Searches pollution and green spaces

**Pubmed**

("environmental health"[Mesh] OR "Environmental Pollution"[Mesh] OR "Environmental Exposure"[Mesh] OR "Air Pollution"[Mesh] OR "Air Pollutants"[Mesh] OR "Water Pollution"[Mesh] OR "Waste Products"[Mesh] OR "Occupational Exposure"[Mesh] OR "Climate Change"[Mesh] OR "Global Warming"[Mesh] OR "Sea Level Rise"[Mesh] OR "Parks, Recreational"[Mesh] OR "green space" OR "Urban Health"[Mesh] OR "Air Pollution"[Mesh] OR "Air Pollutants"[Mesh] OR "Water Pollution"[Mesh] OR "Waste Products"[Mesh]) AND ("Mental Health"[Mesh] OR "Mental Disorders"[Mesh] OR "Anxiety Disorders"[Mesh] OR "Bipolar and Related Disorders"[Mesh] OR "Disruptive, Impulse Control, and Conduct Disorders"[Mesh] OR "Feeding and Eating Disorders"[Mesh] OR "Mood Disorders"[Mesh] OR "Neurocognitive Disorders"[Mesh] OR "Neurodevelopmental Disorders"[Mesh] OR "Schizophrenia Spectrum and Other Psychotic Disorders"[Mesh] OR "Substance-Related Disorders"[Mesh] OR "Trauma and Stressor Related Disorders"[Mesh])

**PsycINFO**

SU environmental health OR SU pollution OR SU climate change OR SU global warming OR SU green space OR SU urban health OR SU pollutants OR SU recreational parks OR SU occupational exposure

Limits, methodology: meta-analysis

Limits, methodology: systematic review

**EMBASE**

‘environmental health’ OR ‘Environmental Pollution’ OR ‘Environmental Exposure’ OR ‘Climate Change’ OR ‘Global Warming’ OR ‘Parks, Recreational’ OR ‘green space’ OR ‘Urban Health’ OR ‘Occupational Exposure’ OR ‘Air Pollution’ OR ‘Air Pollutants’

AND ‘mental disease’

AND ('meta analysis'/de OR 'systematic review'/de)

Supplement 2. Further details for AMSTAR-2 scoring

The quality of the included meta-analyses was assessed with the AMSTAR 2, a critical appraisal tool for systematic reviews (Shea et al., 2017). AMSTAR-2 critically assesses sixteen core characteristics of systematic reviews: (1) an adequate definition of the PICO (participants, intervention, comparator, outcome) acronym when formulating the research question, (2) whether the methods were established before the conduct of the review, (3) an explanation for the selection of study design to be included, (4) the comprehensiveness of the search strategy, (5) study selection by at least two reviewers, (6) data extraction by at least two reviewers, (7) providing a list of excluded studies with reasons, (8) a detailed description of included studies, (9) assessment of risk of bias in included studies, (10) the review reported sources of funding for the included studies, (11) appropriate methods for pooling results of individual studies, (12) assessment of the impact of risk of bias on the outcomes, (13) a discussion of the impact of risk of bias on results, (14) an explanation and discussion of heterogeneity, (15) assessment of publication bias, (16) reporting potential conflict of interest and funding for the review.

Items 1, 3, 5, 6, 10, 11, 12, 13, 14, 15 and 16 were rated as positive (Yes) or negative (No), and Items 2, 4, 7, 8, and 9 also included a probably positive answer (Partial Yes).

We slightly adapted the scoring of item 4 the AMSTAR-2 tool to make it applicable for the included pool of literature. Item 4 refers to the comprehensiveness of the search strategy, and should be rated as partial positive (Partial Yes) when the researchers completed the following requirements: a) searched at least 2 databases, b) provided key word and/or search strategy, and c) justified publication restrictions (e.g. language). For a complete positive rating (Yes), they should also meet all the following requirements: d) searched the reference lists of included studies, e) searched trial/study registries, f) included/consulted content experts in the field, g) where relevant, searched for grey literature, and h) conducted the search within 24 months of completion of the review.

In our umbrella review, since all the meta-analyses were focused mostly on observational literature, we decided to not penalize the reviews based on requirement e) search study registries for the identification of unpublished or ongoing studies, since study registries for observational literature are not as widespread as those for clinical trials. Nevertheless, we acknowledge the importance of registration practices for observational designs. In addition, we did not penalize reviews based on requirement f), and assumed that all meta-analyses contained experts in the field among their co-authors.

Still, even without penalizing studies based on requirements e) and f), only two reviews attained a complete positive rating (Yes) in item 4, given that many of the reviews did not search for grey literature.

Supplement 3. Details of the included meta-analyses on climate events, pollution and green spaces

A total of 13 meta-analyses examined the association between climate events and mental health outcomes. Two meta-analyses were focused on ambient temperature and heat waves and their link to suicide risks (Heo et al., 2021) and mental health-related mortality and morbidity (Liu et al., 2021). The remaining 11 reviews examined natural disasters, with most studies focusing specifically on earthquakes (n=6), reporting on PTSD (Cenat et al., 2020; Dai et al., 2016; Hosseinnejad et al., 2021; Liang et al., 2021; Sepahvand et al., 2019), suicidal ideation (Jahangiri et al., 2020), and depression and anxiety (Cenat et al., 2020) in all age groups. One review examined the association between earthquakes and floods and PTSD in children and adolescents (Rezayat et al., 2020), while another one was focused on floods and reporting as well on PTSD in all ages (Chen et al., 2015). The association between exposure to hurricanes and mental health was examined in one meta-analysis, which was centered on hurricane Katrina and PTSD in adults (Chan et al., 2014). Finally, two reviews explored the link between various natural disasters (earthquakes, floods, hurricanes, tsunamis, wildfires, tornados, etc.) with internalizing and externalizing problems in youth (Rubens et al., 2018) and with psychological distress and various psychiatric disorders (PTSD, depression, anxiety, alcohol abuse) in adults (Beaglehole et al., 2018).

The association of pollution and mental health outcomes was examined in 11 meta-analyses. Most of them examined exposure during pregnancy to air pollution (particulate matter, ozone, nitrogen dioxide, carbon monoxide, etc.) and its potential link to autism spectrum disorder in children (n=4) (Chun et al., 2020; Dutheil et al., 2021; Flores-Pajot et al., 2016; Lam et al., 2016). Four studies investigated the association between exposure to these air pollutants and depression or suicide risk in all ages, but mostly focusing on adults (Heo et al., 2021; Braithwaite et al., 2019; Zeng et al., 2019; Generaal et al., 2019). The exposure to pollutant substances through other sources (e.g., contaminated water, indoor air, or various consumer products such as pan coatings, food packaging, or outdoor gear) was examined in two meta-analyses. One explored childhood exposure to inorganic arsenic and lead and its association with autism spectrum disorder (Wang et al., 2019), and the other study pooled data from nine European cohorts examining maternal exposure to perfluoroalkyl substances and attention deficit/hyperactivity disorder in children (Forns et al., 2020). Finally, two meta-analyses reported on the potential link between noise pollution and mental health.

These specifically examined traffic noise and its potential association to depression and/or anxiety in adults (Hegewald et al., 2020; Generaal et al., 2019).

Finally, we identified two meta-analyses reporting associations between exposure to green spaces or natural environments and depression. One of them focused on short-term exposure to natural environments (e.g., forests, urban and natural parks) and its link to depressive mood (Roberts et al., 2019), while the other analyzed the association between green spaces and urbanization and depression (Generaal et al., 2019).
